# Crude oil recovery by new nonionic surfactants in relation to equivalent alkane carbon number (EACN) with work adhesion and alteration wettability

**DOI:** 10.1038/s41598-025-99993-8

**Published:** 2025-05-17

**Authors:** A. M. Al Sabagh, Ahmed I. Khodair, B. M. El-Sadek, Entsar E. Badr, Esraa Hamada Kalboush

**Affiliations:** 1 Application Department, Egyptian Petroleum Research Institute (EPRI), Nasr City, Cairo Egypt; 2 Chemistry Department, Faculty of Science, KafrelSheikh University, KafrelSheikh, 33516 Egypt; 3 Chemistry Department, Faculty of Science, Al- Azhar University (for Girls), Nasr city, 11754 Cairo Egypt

**Keywords:** Hydrazide surfactants, Nonionic surfactants, Alkane carbon number, Interfacial tension, Work adhesion, Contact angle, Spreading coefficient, Chemistry, Energy science and technology

## Abstract

Six new nonionic surfactants abbreviated as (PMRH x), besides cationic surfactant ( RHATAS), based on ricinoleic hydrazide were prepared elsewhere. The nmin and alkane carbon number (ACN) at 50°C and concentration of CMC were determined by the n-hydrocarbon scans (n-c_6_ to n-c_18_) against the tension between n-c and surfactant solution. The work adhesion (Wa), surface charge energy, and spreading coefficient were calculated for these surfactants based on surface, interfacial tension, and measuring of contact angle. The rheological properties of the solution of these surfactants at CMC were measured at 50 °C to determine the dynamic (η_app_) and plastic viscosities (η_pl_). Otherwise, the recovery factor of the crude oil was investigated by using a sand back model at 50 °C using waxy crude oil. The effect of ethylene oxide content in the surfactant structure was discussed via all the studied parameters. The surfactant PMRH 136 exhibited the maximum oil recovery (85.20%) individually, and its blend with RHATAS achieved a recovery factor (92%). A mechanism to describe the effect of these parameters on the recovery factor is introduced in the text.

**Supplementary Information:**

The online version contains supplementary material available at 10.1038/s41598-025-99993-8.

## Introduction

Global energy demand is predicted to rise by 30% between 2010 and 2040^1^. Additionally, it is projected that oil consumption will reach 111.1 million barrels per day by 2040^2^. Improving oil recovery from diminishing oil reservoirs has become more essential due to the depletion of oil supplies and the rise in energy demand caused by population expansion and rapid industrial development^3,4^. Crude oil recovery can be divided into three main stages: primary, secondary, and tertiary. The first and secondary stages of oil extraction are known as traditional methods in the petroleum industry .The third phase, however, is known as enhanced oil recovery (EOR)^5^. Less than 30% of original oil in situ (OOIP) is produced by the primary recovery using artificial lift and natural flow^5,6^. Oil is pushed out of the petroleum reservoir by the natural pressure of trapped crude oil. As the main process proceeds, the reservoir pressure decreases below a certain level, which prevents the trapped oil from pushing in the direction of producing wells. Gas or water injections are used in secondary oil recovery to preserve or improve the natural pressure in the reservoir^7^. In water flooding, a reservoir’s pressure is maintained by injecting water into it through a series of injection wells^3^. The projected amount of oil recovered in the primary and secondary processes typically equivalent to 20–50% of the deposit, depending on the characteristics of the oil and reservoir^8^. Because of this, the residual oil in the reservoir can be extracted using a tertiary recovery phase, commonly referred to as enhanced oil recovery (EOR). Among the several processes involved in EOR is chemical flooding^9,10^. Gas injection^11,12^ ,and thermal recovery^13^. Gas injection techniques use a variety of gases to improve oil recovery. The advantages of gas injection include improved oil recovery, extra reservoir life, and increased overall oil extraction process efficiency^14^.The effectiveness of the surfactant is determined by lowering the interfacial tension to very low levels (as low as 10^–4^ mNm^− 1^), between the injection fluid and the recovered crude oil that is still in the reservoir^15,16^. Maintaining a low IFT for prolonged periods of time is necessary for surfactant flooding to be effective^17^. Oil recovery must be increased by using new recovery techniques because the rate of oil production from reservoirs has decreased in the recent decades^18,19^. One application for emulsions is emulsion flooding for enhanced oil recovery (EOR). The EOR method involves combining water and crude oil to create an emulsion, which is then stabilized by adding surfactants. The main objective of the additional surfactants is to target the ultralow of interfacial tension (10 ^− 2^ 10 ^− 4^ mNm^− 1^). At the same time, the alkane carbon number (ACN) of the crude oil, which varies from 7 to 9, must be attained in order to achieve the lowest possible interfacial tension^20,21^. Early studies on EOR in the literature also indicated that the alkane carbon number (ACN (, or the number of carbons in a straight alkane chain) for normal alkanes, or so-called )EACN( for a mixture of hydrocarbons or non-alkyl hydrocarbons, was largely related to the optimal brine salinity developed for a particular brine-oil-surfactant mixture^22–25^. The alkane model, first presented by Cayias,^25^ greatly facilitates the study of interfacial tensions in an oil-surfactant-water system. In order to study the interfacial tensions of surfactant systems against n-alkanes, Cayias introduced the idea of the equivalent alkane carbon number^26,27^. This model resembles a) V-shape (of the relationship between the interfacial tension and the n-alkanes (n-C6 to n-C18). The Cayias model is used to determine the minimum alkane carbon number (n _min_) at which the interfacial tension is reduced to its greatest extent. The interfacial tensions between surfactant solutions with varying )n _min_ ( values and different model oils with different equivalent alkane carbon number (EACN) values have also been determined^28^. The hydrophobicity of crude oil is represented by )EACN(, making it a crucial parameter for finding out the ideal salinity. Knowing the )EACN( of the crude oil makes it easier to formulate a middle phase micro emulsion system (concurrent with ultra-low IFT value under optimal Salinity condition) for EOR by surfactant flooding. Thus, before creating a surfactant formulation for EOR, it would be beneficial to ascertain the )EACN( of the crude oils. When created with true ternary s-o-w (surfactant-oil-water) systems, the properties of micro- and macro-emulsions depended on both the oil and the surfactant’s nature^27^. Several parameters’ effects on )n _min_ (have been previously reported and briefly reviewed. Currently recognized as critical factors in achieving low interfacial tension are the electrolyte, surfactant concentration, surfactant average molecular weight, the type of oil phase age of the surfactant solution, and alcohols^29,30^. The nature of the oil, which is frequently expressed by the alkane carbon number (ACN) or the equivalent alkane carbon number (EACN) in cases where the oil phase is not an alkane, is one of the formulation variables^31^. When the hydrophobic alkyl group chain length is increased for a succession of surfactants with the same basic molecular structure, (n _min_) increases almost linearly; hence, the equivalent alkane carbon number (EACN) increases as the molecular weight increases. However, the (n _min_) is decreased when hydrophilic groups, such ethylene oxides, are added to increase the molecular weight. In fact, it appears to be better to modify (n _min_) by adjusting the hydrophilic = lyophilic balance (HLB) of the surfactant^32^. In addition to the surfactant molecular weight, the hydrophobicity of anionic surfactants can be modified by altering the hydrocarbon group structure. Some researchers have studied this significant impact of the molecular structure on n _min_^32^. It has been demonstrated in more recent research that anionic extended surfactants, which contain intermediate-polarity groups like propylene oxide (PO) and ethylene oxide (EO) inserted between the hydrophilic head and hydrocarbon tail of the surfactant, can produce middle phase micro-emulsions with low IFT and high solubilization of oil without compromising their water solubility^33^. As a result, several extended surfactants with various characteristics recently made a lot of attention and been studied for numerous practical applications because of these appealing qualities that are very desirable for practical uses^34–36^. Even so, the (EACN) of crude oils has only been the object of a small number of research^37^ .

The main object of this work is to use these surfactants in two forms, nonionic and cationic moieties, in the chemical EOR application to evaluate their potential in the enhanced recovery factor (RF). Attention should be extended to studying the different parameters, alkane carbon number, IFT, and contact angle, to calculate the work adhesion, spreading coefficient, and surface distribution charge and investigate their effect on the RF. A mechanism of wettability alteration should be introduced in the light of these standard parameters.

## Material and measurements

### Material

#### Surfactants

Seven surface-active agents were prepared based on ricinoleic hydrazide. The six surfactants that have a nonionic moiety were prepared by a reaction of ricinoleic hydrazide with succinic anhydride in an open-ring reaction, and then the product reacted with different molecular weights of polyethylene glycol to produce this formula (PMRH x). The letter seven surfactant has a cationic moiety, and its general formula is (RHATAs)^38^.

#### The used crude oil and formation water

**Fig. 1:**
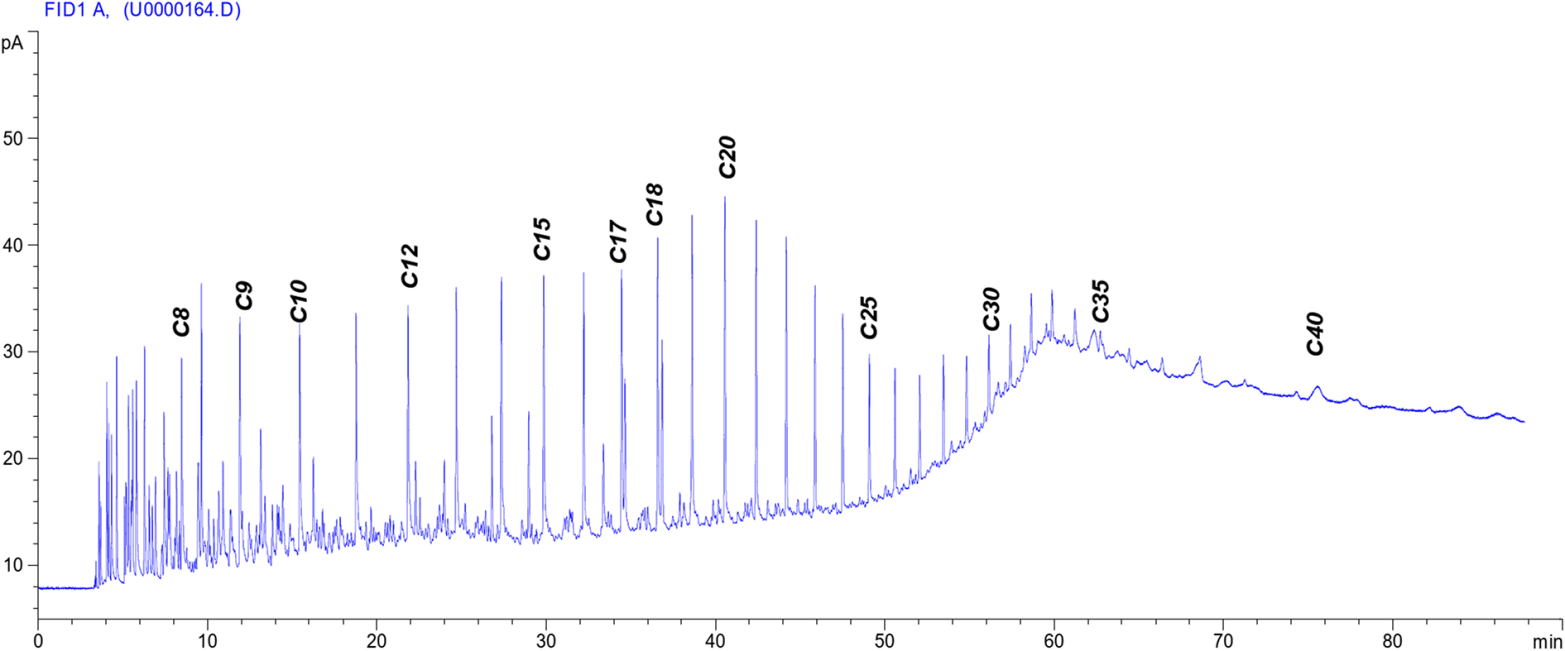
The GC chromatogram of the used crude oil.

The crude oil was supplied by the General Petroleum Company (GPC), Egypt. The physical and chemical properties are shown in Table 1. Figure 1 describes the gas chromatography for the used crude oil to determine its equivalent alkane carbon number (EACN).

The formation water was also supplied from the (GPC), Egypt. Its TDS was 50 × 10^3^ ppm.

**Table 1:** General characterization of the used crude oil.

Specification	Method	Result
Density @ 15.56 °C Specific gravity API gravity @ 60 °F	ASTM D-5002 ASTM D-4052 ASTM D-5002	0.8537 0.8546 34.08
Kinematic viscosity, cSt, @ 40^o^ C	ASTM D-445	5.890
Asphaltene content, wt%	IP-143	5.900
Wax content, wt%	UOP-64	15.50
Pour point, °C	ASTM D-97	9.000
Flash point, °C	ASTM D-93	˂30.00
Water content, vol%	ASTM E-203	0.000

### Measurements

#### Surface and interfacial tension measurement

The sessile drop technique was employed by a Theta optical tensiometer to measure the tension between surfactant solution and oil ( IFT ) at 50 °C in formation water^39–41^. The pendent drop technique was employed by a Theta optical tensiometer to measure the surface tension of surfactant solution at different concentration in formation water. The CMC is the concentration at which surfactant molecules start to form micelles in the solution by absorbing more of the interface than a monolayer. The interfacial tension was measured for the blank sample (used crude oil, 24.5mNm^− 1^).

#### Contact angle measurement

The wettability of a flat rock surface saturated with crude oil from the reservoir model was evaluated by measuring the contact angle. The sessile drop method was used to conduct the experiments at 50 °C using a Theta optical tensiometer^41^. At the critical micelle concentration (CMC), the contact angle was measured with and without the surfactants.

#### Dynamic viscosity measurement

The PVC Brookfield was utilized to measure the dynamic viscosity for untreated and treated crude oil with the used surfactants at concentrations of the CMC and 25 and 50 °C. The Yield value ƮB, apparent (η_app_), and plastic (η_Pl_) viscosity values were determined. The flow curves can be analyzed using the Herschel-Bulkley equation:

1 $$\k{T}= \k{T}{_B} + {\rm{K}}\,{\rm{DM}}$$

Where Ʈ represents the shear stress ƮB represents the dynamic yield stress “Bingham yield value”, K represents the consistency index, m represents the shear thinning index and D represents the shear rate. The Bingham yield value (ƮB) may be derived from the graph`s intercept showing the relationship between shear stress and shear rate. However, the dynamic apparent viscosity is obtained from the straight line of the shear rate – viscosity equation. The slope of the lines between shear rate and shear stress is expressed.

#### Surfactant flooding Test^42,43^

**Fig. 2:**
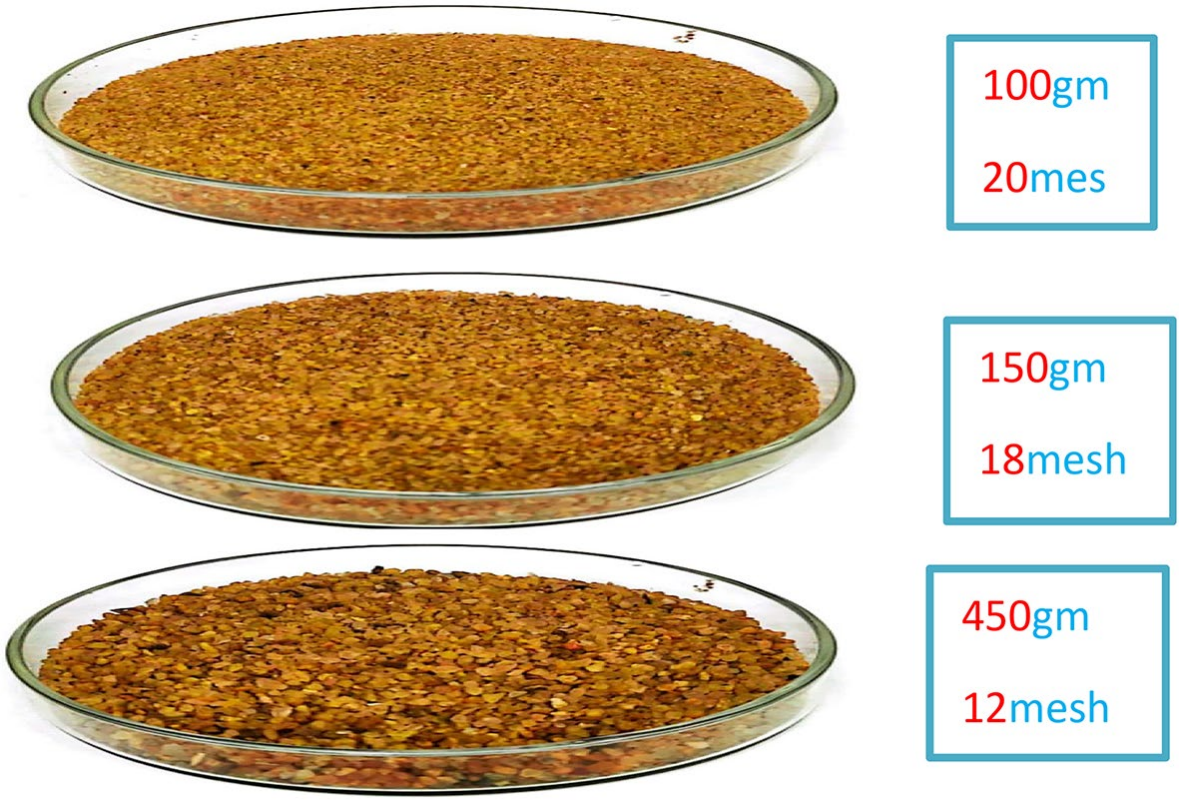
Different sizes of the used sand for packing the sand stone model.

In this experiment, sand-packed equipment was used to perform a surfactant flooding test. The apparatus measured 30 cm in length and 5.0 cm in internal diameter, yielding a bulk volume of 589.28 cm^3^. The model was filled using sand of varying sizes. To achieve a specific porosity of 23.75%, the sizes utilized were 12, 18, and 20 mesh (1.68, 1.00, and 0.841 mm), respectively. Figure 2 shows the various kinds and sizes of sand that were utilized to create the sand-packed model. The apparatus was saturated for two days with formation water before the experiment. Then, at a rate of 1 cm^3^/min, 140 cm^3^ of oil was injected into the apparatus while the reservoir was at 50 °C and 2.0 MPa. 120 milliliters of crude oil out of 140 milliliters saturated the model; this amount (130 milliliters) is known as the initial oil saturation volume (VOI) and may also be referred to as the original oil in place (OOIP). The initial water saturation volume, or VWI (the volume of formation water left in the model after applying the oil saturation), is the remaining 10 ml from the pore volume. After that, the oil had been stored at 50 °C for 24 h. The amount of oil recovered during the secondary recovery stage was then evaluated by water flooding with formation water (TDS: 50,000 ppm). The tertiary oil recovery was then carried out, using concentration at CMC of the used surfactants, during the performing flooding process (3ry recovery).

The following steps were taken to complete the core-flooding experiments^44–47^.

1. **Packing of sand (Sand stone rock Model) to get porosity**

2 $${\rm{Pore\, Volume = }}{{\rm{V}}_{{\rm\textbf{total\,distilled\, water\, used\, in\, Packing}}}}{\rm{ = 140 ml}}$$

The sand-pack model was cylindrical, with 30 cm in length and 5.0 cm in internal diameter. So, the volume of the sand pack can be calculated as follows:

So, the total bulk volume is equal to 589.28 cm^3^.

Hence, the porosity can be determined:

3 $${\rm{Bulk\, Volume = \pi }}{{\rm{r}}^{\rm{2}}}{\rm{h\;}}$$

4 $${\rm{Porosity = Pore\, Volume / Bulk\, Volume}}$$

The porosity of the sand is then equal to 23.75%.

2. **Formation water saturation**

Formation water was injected until pronouncing complete saturation.

3. **Oil saturation**

After saturation of the sand pack by formation water, the crude oil of 40 cP was injected at a constant rate of 10 ml/h through the sand pack to calculate the original oil in place (OOIP).The core was aged for six days to achieve a laboratory-scale petroleum reservoir model .To calculate the initial water saturation and subsequently the initial oil saturation (OOIP), the volume of crude oil saturated the model was measured by measuring the displaced water after oil saturation.

From the 1st step, the pore volume is equal to 140 ml.

From this 3rd step: the displaced water after oil saturation is equal to 130 ml. From this value, the original oil in place (OOIP) can be calculated as follows:

5 $${\rm{Initial\, water\, saturation\, volume,\,}}{{\rm{V}}_{{\rm{WI}}}}\left( {{\rm{ml}}} \right){\rm{ = Pore\, Volume - Displaced\, Water}}$$

6 $${\rm{Initial\, Water\, Saturation\, Percent,\,}}{{\rm{V}}_{{\rm{WI}}}}\left( {\rm{\% }} \right)\:\left( {\frac{{{\rm{Initial}}\:{\rm{Water}}\:{\rm{Saturation}}\:{\rm{volume}}\:\left( {{\rm{ml}}} \right)\:}}{{{\rm{Pore}}\:{\rm{Volume}}}}} \right){\rm{x}}\:100$$

As the displaced water = 130 ml.

So, the initial water saturation volume (ml) = 140–130 = 10 ml.

Initial water saturation % =$\:\:\left(\frac{10}{140}\right)\text{x}\:100=7.14\:\text{\%}$

Then the original oil in place can be computed.

7 $${\rm{OOIP = \;Is\, the\, initial\, oil\, saturation\, volume,\,}}{{\rm{V}}_{{\rm{OI}}}}\left( {{\rm{ml}}} \right){\rm{ = Pore\, Volume\,-}}{{\rm{V}}_{{\rm{WI}}}}\left( {{\rm{ml}}} \right)$$

8 $${\rm{Initial\, oil\, saturation\, percent,\,}}{{\rm{V}}_{{\rm{OI}}}}\left( {\rm{\% }} \right){\rm{ = }}\:\left( {\frac{{{\rm{Initial}}\:{\rm{Oil}}\:{\rm{Saturation}}\:{\rm{volume}}\:\left( {{\rm{ml}}} \right)\:}}{{{\rm{Pore}}\:{\rm{Volume}}}}} \right){\rm{x}}\:100$$

Therefore, OOIP = Is the initial oil saturation volume, **V**_**OI**_ (ml) = 140–10 = 130 ml.

Initial oil saturation Percent (**V**_**OI**_, %) = $\:\left(\frac{130}{140}\right)\text{x}\:100=92.85\:\text{\%}$

4. **Secondary oil recovery (Formation water Flooding)**

The formation of water flooding as a secondary oil recovery system was performed with a fixed flow rate of 3 ml/min. Formation water was constantly pumped until the sand pack model stopped producing any oil (or less than 1 ml). The oil and water which were produced as a result of the flooding were collected in the various graduated cylinders. The volumes of oil gathered have been recorded and this is the recovery factor volume of the secondary stage (**RF2ry**, ml). From this value, the recovery factor percent of the secondary recovery (**RF2ry**, %) can be calculated. The water breakthrough has also been recorded. After the formation water flooding process was completed, the residual oil saturation “**SOR**” (which should be produced by the 3ry recovery) was also determined. The residual oil saturation volume of the secondary recovery, denoted as “**SOR (2ry)**,** ml**” represents the volume of oil that is left within the rock after completing the brine flooding. Subsequently, the residual oil saturation percent “**SOR (2ry)**,** %**” can then be computed.

5. **Tertiary oil recovery by surfactant flooding**

To simulate the reservoir temperature, the sand pack model’s temperature was adjusted to 50oC. The surfactant solution was flooded at a rate of 3 ml/ min after the sand pack model temperature had been raised to 50°C. The surfactant solution above the CMC concentration in formation water was injected until no further oil was released from the model. The liquid (oil and water) recovered by displacement process, was collected in the effluent collector or different graduated cylinders. The tertiary oil recovery volume (**RF3ry**,** ml**) and subsequent percentages (**RF3ry**,** %**) were determined at the end of this stage.

By utilizing the data on (RF2ry, ml) and (RF3ry, ml), it is possible to compute both the overall recovery and the residual oil content following the implementation of secondary and tertiary recovery processes^46,48–50^

9 $${\rm{The\, Total\, Recovery = \textbf{(R}}{{\rm\textbf{F}}_{{\rm\textbf{2ry}}}}{\rm\textbf {, ml) + \textbf{( R}}}{{\rm\textbf {F}}_{{\rm\textbf {3ry}}}}{\rm\textbf{ {,ml})}}}$$

10 $${\rm{The\, Residual\, Oil }} = {\bf{OOIP}}-[({\bf{R}}{{\bf{F}}_{{\bf{2ry}}}},{\rm{ }}{\bf{ml}}){\rm{ }} + {\rm{ }}({\bf{R}}{{\bf{F}}_{{\bf{3ry}}}},{\rm{ }}{\bf{ml}})]$$

The produced oil and water from these surfactant displacements run, with different slug sizes, were collected in different graduated cylinders. The produced oil volumes have been reported for each injected surfactant slug size. Furthermore, the water breakthrough for these different surfactant slug sizes has been recorded. The properties of the reservoir model and condition are shown in Table 2^51^.

**Fig. 3:**
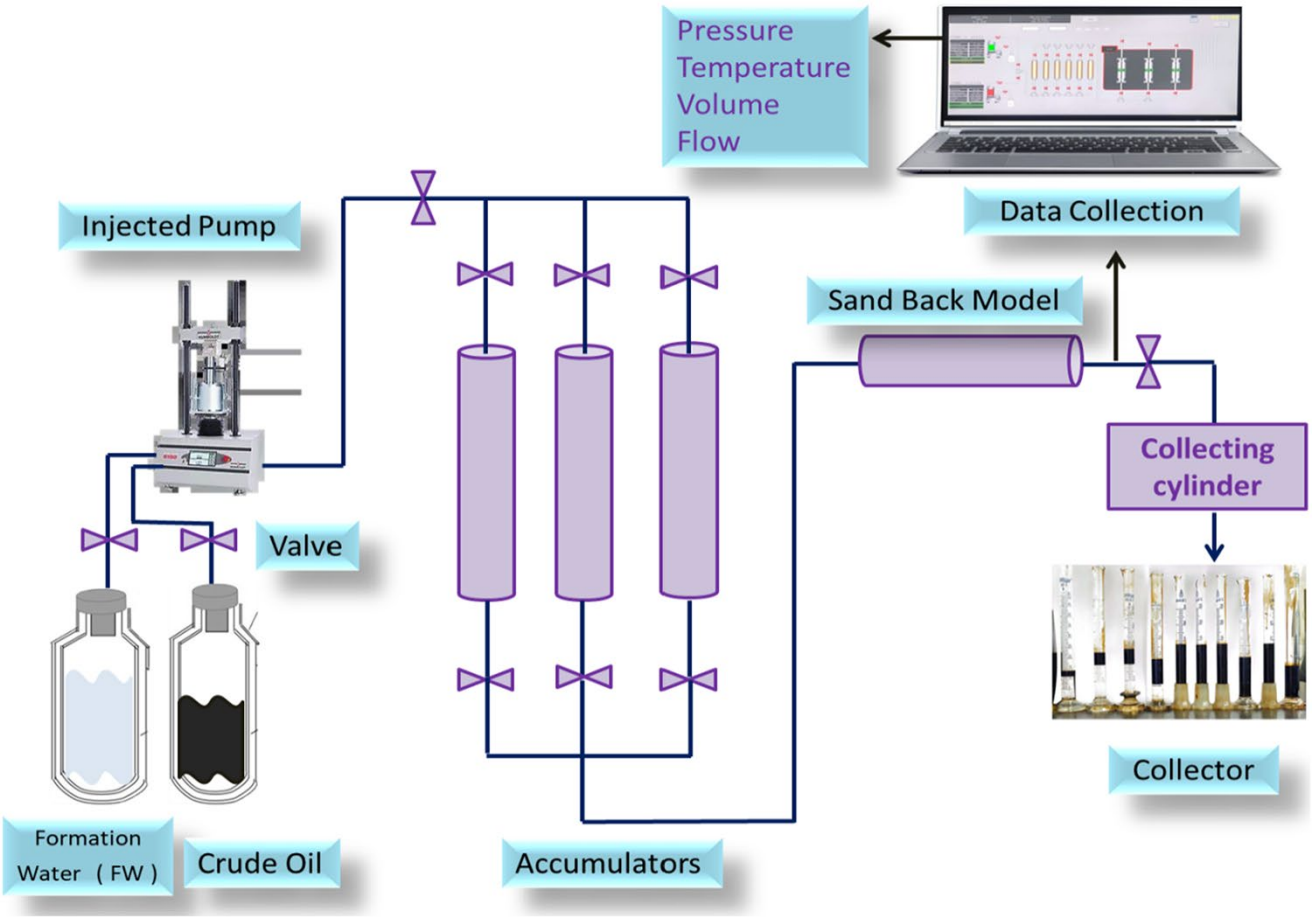
Chemical flooding flow chart.

The chemical flooding system used to perform the flooding test is depicted schematically in Fig. 3. The main components of this device include three cylinders containing formation water, crude oil, and chemical solution in the formation water, an injection pump, plug holder, back-pressure pump, production fluid collection cylinder. To control the temperature, the cylinders, and the plug holder are housed inside an oven. For the injection of certain fluid, the values in the connection line between the cylinder holding the other fluids are closed, whereas, the other valve was opened with the other injecting fluid. The injection rate was set to 2 cc/min.

**Table 2:** Properties of the reservoir model and condition.

Property	Value	
Sample type	Sandstone	
Diameter(mm)	50.0	
Length (mm)	300.0	
PV Known (cc)	140.0	
ρfluid (g/cc)	1.0	
Porosity (%)	23.0	
Reservoir Pressure, psi	Injection	Up to 500
	Back Pressure	Up to 200
Reservoir Temperature ,°C	50	

## Results and discussion

### The tension between surfactant solution and hydrocarbon system (ACN)

**Fig. 4:**
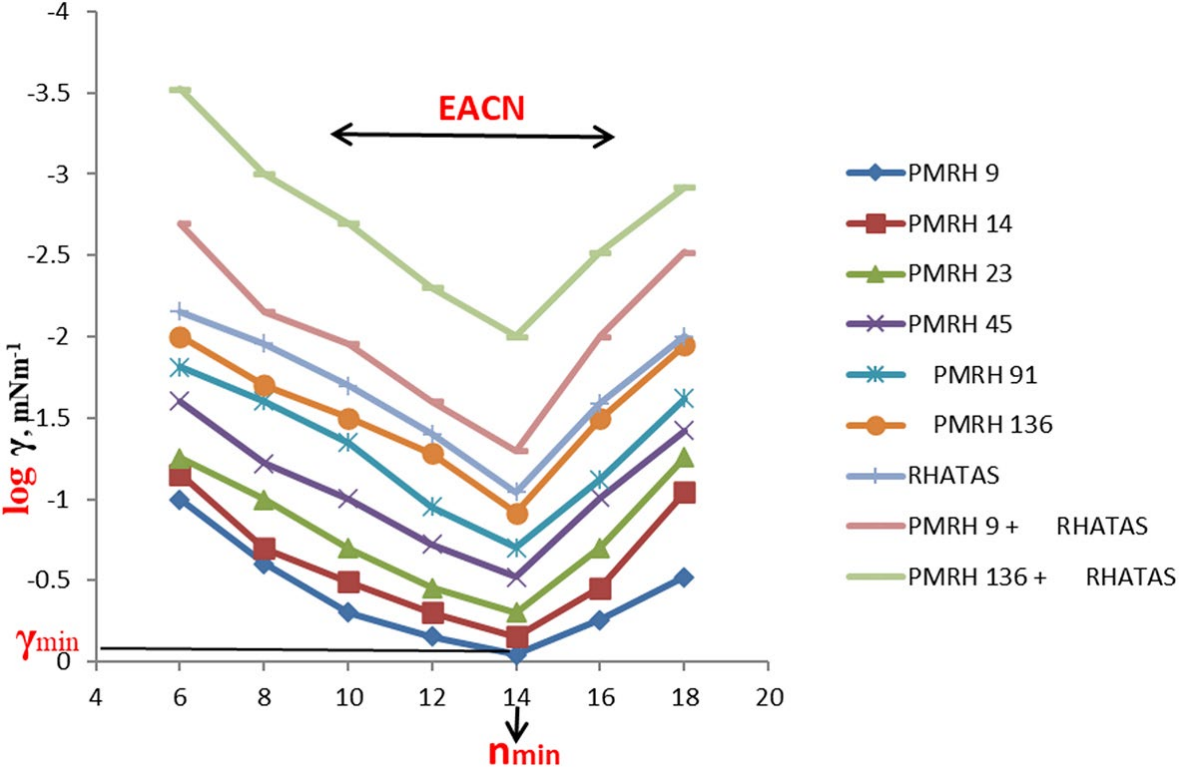
The ( n _min_ )and Equivalent Alkane Carbon Number of the Used Surfactants and Oil.

The interfacial tension between crude oil and the injection chemical plays a vital role in the oil recovery process. The undertaken surfactants can reduce the interfacial tension between crude oil and its aqueous solution phase by increasing the ethylene oxide number in the molecule^52^, as shown in Table 3. The reduction of IFT is a good indication for the formation of water base emulsion (O/W) during the surfactant flooding process, which will help achieve the best possible oil recovery. The IFT was determined against n-alkanes to detect the (n _min_) (at which the minimum IFT was achieved). It can be identifying the EACN from the V-shaped curves as shown in Fig. 4. The equivalent alkane carbon number (EACN) is shown on the curve lines in Fig. 4. This means that every used surfactant achieved a certain (n _min_) at n-c14, but at the same time by some different ratio of different n-alkanes up and down the (n min ) can detect the equivalent alkane carbon number (EACN) of the used crude oil. If the used surfactant exhibited a minimum tension in the range of the EACN, it should be pronounced an emulsion (O/W) during the EOR process. By analysis of the GC chromatogram (Fig. 1.), it was found that the total molecular weight of the wax and paraffin of the used crude oil was about 14 (the main soluble hydrocarbon). This foundation is very important to detect the (n _min)_ and the EACN of the used surfactant with the used oil. These factors are considered promising parameters during an investigation of surfactants in chemical oil recovery. The surfactant in this study exhibited (n _min_ ) at n-c14. The γ_min_ (the minimum interfacial tension at the (n _min_) was sensitive to the ethylene oxide number (EO). The γ_min_ was measured by an increase of the EO number as shown in Fig. 4. The PMRH136 exhibited the minimum value of γ_min_ (7 × 10^− 2^ mNm^− 1^) at n-c14. The more minimum IFT was obtained by the blend of PMRH136 with RHATAS (4 × 10^− 2^ mNm^− 1^).

**Table 3:** Surface tension, interfacial tension and contact angle of the blank oil, used surfactant and their some blends in the formation water at CMC concentration.

Surfactant	IFT (mNm^− 1^)	Surface Tension (mNm^− 1^)	Static Contact Angle (θ)
Blank Oil (Used)	-----	------	159˚
PMRH 9	9 × 10^− 1^	32.31	41
PMRH 14	7 × 10^− 1^	33.11	36
PMRH 23	5 × 10^− 1^	37.21	30
PMRH 45	3 × 10^− 1^	37.53	25
PMRH 91	1 × 10^− 1^	37.86	19
PMRH 136	7 × 10^− 2^	38.34	12
RHATAS	9 × 10^− 2^	35.78	22
PMRH 9 + RHATAS	5 × 10^− 2^	34.33	15
PMRH 136 + RHATAS	4 × 10^− 2^	35.67	12

### Contact angle and wettability alteration

One important feature that surfactants can provide in a variety of applications is the capacity to alter surface wettability. Enhancing the effectiveness of oil recovery (EOR) requires changing the wettability of reservoir rocks in petroleum wells^53^ The hydrophilicity of the surfactants used for oil displacement is higher than the lipophilicity. When these surfactants are adsorbed on the surface of the formation rock, they can change the rocks’ wettability, which lowers the contact angle between the rock surface and the formation water. This effect increases the efficiency of displacement and decreases the adhesion work between rock and oil. It is expected that water-wet reservoirs will be a good source for obtaining high oil recovery. By measuring the contact angle, θ, with either water or oil droplets, one can determine the strength of the attraction between the water and oil phases in the three-phase system (water solution, oil, and rock surface). In general, a system is categorized as water-wet when using water droplets if the contact angle is between 0˚ and 90˚. While contact angles above 100˚ represent oil-wet conditions, those between 90˚ and 100˚ represent an intermediate wet regime. Thus, a contact angle of less than 90˚is usually required when using the produced surfactants to improve chemical EOR (Enhanced Oil Recovery)^54^.

The arising different equilibrium phases of the contact angle are associated with the free interfacial energy. This was explained by some investigators^55^. The surfactant in the formulation of EOR plays an important role in reducing the tension between the formation water and tested oil, consequently, affecting the contact angle between oil and water at the interface area and making an emulsifier to solubilize the oil in the form of emulsion phase (O/W). So, if the angle of contact in the hydrocarbon system is < 90˚ the oil surface is concave toward the water producing a W/O (water in oil emulsion). On the other hand, if the contact angle is measured in the water phase and gives a value < 90˚, the water surface is concave toward the oil to produce an O/W (oil–in–water emulsion). The type of O/W emulsion is very preferable in the EOR application because it indicates the surfactant flooding carried out as it was expected, as shown in Table 3. The data in Table 3, indicate that the used blank oil droplet’s contact angle was 159˚ in formation water, which is noticeably high. The sand model sample thus changes from wetted oil phase to the aqueous phase when the surface charge (γ_s_) changes from negative to positive value. The surface free energy of the used surfactants was positive values as shown in Table 4. The γ_s_ increased by EO increase. The minimum γ_s_ was obtained with PMRH 9 (0.66 mJ/ m^2^), meanwhile, the maximum of γ_s_ was achieved with PMRH 136 (44.64 mJ/ m^2^). This result may be due to the increase of the (-O-) bond on the poly oxy alkylene chain acts to rises of γ_s_ as the presence of a loin pair of electrons on the oxygen atom. The surfactant decreased the contact angle of oil droplets and interfacial tension at the same time as a result of the decrease of the work adhesion. This finding may explain why the reduction requires work of adhesion to repel the oil droplets away from the rock surface and release them in the form of an emulsion^56–58^.If The contact angle of the water droplet was greater than 100 it was θ = 130.07˚ as in Table 3., suggesting that the rock sample was oil-wet and hydrophobic. The contact angle, however, dropped when the surfactant was added to the formation water at or above the increase of the critical micelle concentration (CMC), signifying that the tested model changed from an wetted oil to an aqueous phase.

These variations in the contact angle and interfacial tension suggest a reduction in the capillary forces, which are responsible for passing the liquid through the openings of the small pores. By lowering the energy required for adhesion, the reduction of capillary forces is essential for improving the oil recovery. Consequently, this facilitates the process of removing oil droplets from the sand model surface and releasing the hydrocarbon as an emulsion phase. The efficiency of oil recovery in the reservoirs that naturally have a preference for oil (oil-wet reservoirs) can be increased by changing the wettability of porous reservoir rocks to become more water-attracted (water-wet). By making this change, the contact angle value (θ) is guaranteed to stay at or below 90˚ degrees (θ < 90˚)^42^. The undertaken surfactants exhibited contact angles as; 41, 36,30, 25, 19, and 12 against the PMRH 9, PMRH 14, PMRH 23, PMRH 45, PMRH 91, and PMRH 136 respectively. While the contact angle of the cationic surfactant was 22˚.When the anionic surfactants were blended with the cationic, the contact angles became 15˚, and 12˚, for the blends (PMRH 9 + RHATAS) and (PMRH 136 + RHATAS) respectively. This change in the contact angle indicates to shift in the phase from wetted oil phase to the aqueous phase of the rock sample. This reduction in the contact angle facilitates the displacement during the oil recovery process .With the increase in the ethylene oxide content. The decrease in the contact angle as shown in Table 3, indicates improved surface wettability. This decrease in the angle of contact may be attributed to the affinity of the used surfactant to reduce the interfacial tension between the water and the oil at rock pores and surfaces. As a result, these surfactants exhibited enhanced wetting properties.

### Work adhesion

In many surfactant applications, the wetting process is a critical character. The reduction of surface tension brought about by a surfactant has an impact on the contact angle (˛θ) of a liquid with a solid surface (oil, formation water, and rock). The contact angle is also affected by the solid surface energy and solid-liquid interfacial tension in addition to the tension of the liquid. The position and orientation of surfactant molecules at the solid-liquid interface have an impact on the adhesion process. The variation between cohesion work (W_c_) and the work of adhesion of solution to the surface (W_a_) can be used to inform the wetting process. The Adhesion work (W_a_) and contact angle (θ) are dependent on the surface tension of both liquid and solid surface tension. The residual oil saturation dropped and the displacement mechanism became simpler as a decrease of contact angle between the fluid, oil, and rock system. For the surfactant molecules present in the flooding process to adsorb in the oil/rock interface layer, the contact angle increases with the oil and decreases with the surfactant liquid/rock. The amount of oil collected increased as a result of the oil starting to flow on the surface of the sandstone or in the pores and becoming soluble as an oil-in-water emulsion. The adhesion work was computed and reported in Table 4. The Younge-Dupre equation was used to compute the work adhesion.

11 $${W_a} = {\gamma _L}\left( \rm{{Cos\theta}+ 1} \right)$$

The young-Dupre equation, which links the work adhesion to the wetting liquid`s surface tension, invariably result in more adhesional wetting ( γ_L_) and contact angle at the solution interface ( θ )^59,60^. From the data in Table 3, it was found that the increase of the contact angle (decrease of Cos θ) reflects an increase in the solution surface tension. When the contact angle is 180˚, which is never reached, the driving force of adhesional wetting is equal to zero and can never be negative. The term “cohesion work” (W_c_) refers to a liquid self-adhesion.

12 $${{\rm{W}}_{\rm{C}}}{\rm{ = 2}}{{\rm{Y}}_{\rm{L}}}$$

When, if W_a_ > W_c_, the spreading coefficient forms a thin layer on the sandstone by spreading on its own, if W_a_< W_c_, the spreading coefficient is negative, if θ is greater than zero, and the liquid produce droplets or lenses with a restricted contact angle rather than spreading across the substrate (sandstone). The spreading coefficient is zero when the work of adhesion and the work of cohesion are equal.

**Fig. 5:**
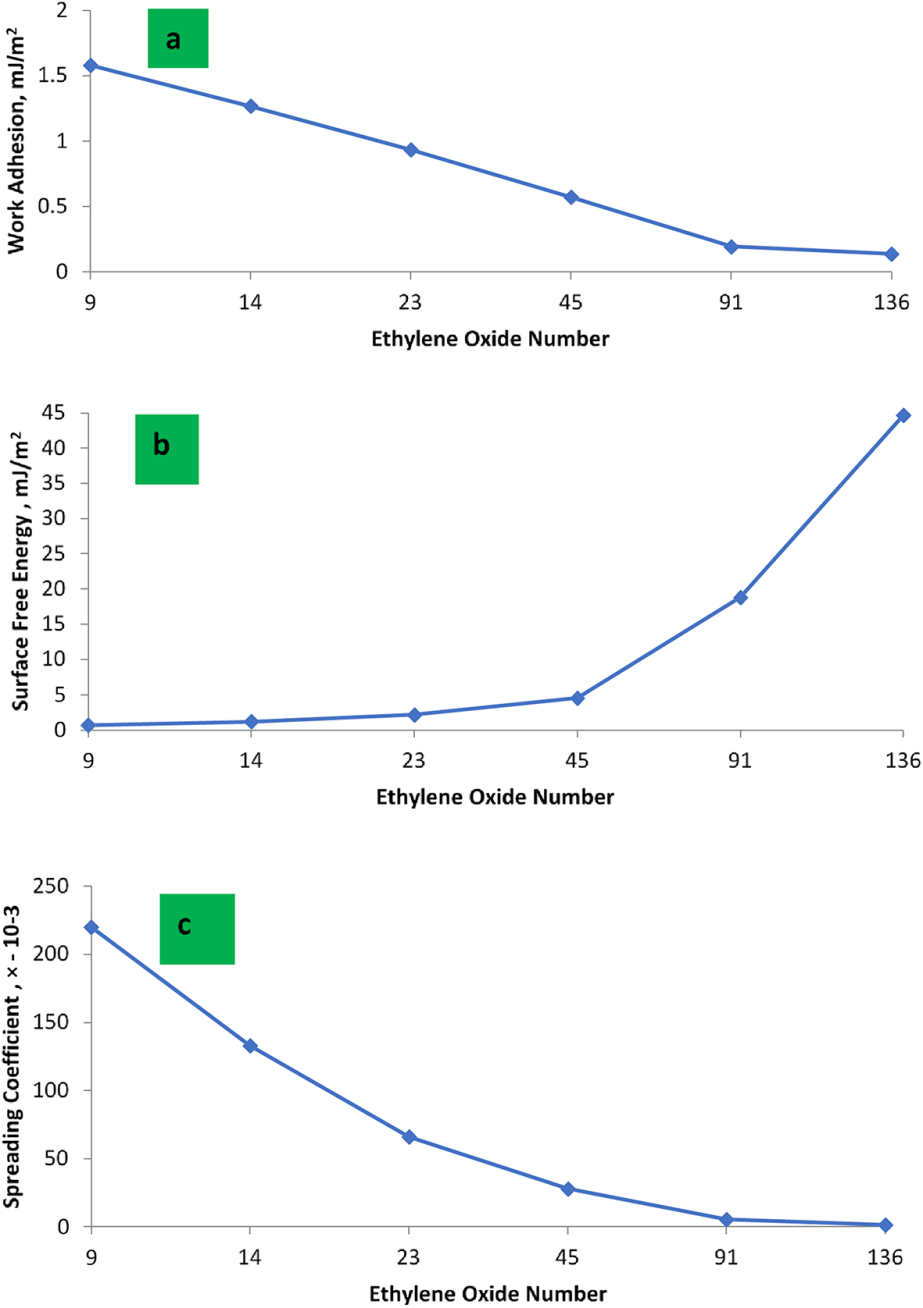
(**a**)-Relation between ethylene oxide number and work adhesion. (**b**) Relation between ethylene oxide number and surface free energy. (**c**) Relation between ethylene oxide number and spreading coefficient.

From the data of (W_a_) in Table 4, it was found that the W_a_ decreases as the EO number increases as shown in Fig. 5. The value of W_a_ for PMRH 9 was 1.579 mJ/m^2^, whereas it was 0.138 mJ/m^2^ for PMRH 136. The blend between the cationic surfactant and nonionic (PMRH 136) exhibited positive synergism and pronounced W_a_ equal to 0.079mJ/m^2^. It seems that the EO content plays an important role in decreasing the W_a_ which eases to repel the oil droplet from the surface.

**Table 4:** Work adhesion, surface free energy and spreading coefficient at 50 °C.

Surfactant	Work Adhesion, W_a_ ( mJ/m^2^)	Surface Free Energy, γ_s_ ( mJ/m^2^ )	Spreading Coefficient, W_s_
PMRH 9	1.579	0.660	-0.22000
PMRH 14	1.266	1.159	-0.13300
PMRH 23	0.933	2.148	-0.06600
PMRH 45	0.571	4.535	-0.02800
PMRH 91	0.194	18.84	-0.00540
PMRH 136	0.138	44.64	-0.00150
RHATAS	0.173	16.75	-0.00650
PMRH 9 + RHATAS	0.098	44.21	-0.00170
PMRH 136 + RHATAS	0.079	72.68	-0.00087

### Spreading coefficient of surfactant on the rock surface

The spreading coefficient, which is a mathematical expression for the variation in the adhesion work and the work of cohesion, describes how liquid spreads over a solid surface. For the oil to naturally spread across the sandstone rock`s surface (or model), reduce the system`s surface free energy, which depends on a decrease in the area at the interface. The saturation of the rock by oil is in equilibrium with interfacial tension. Spontaneous spreading is possible if the spreading coefficient is positive and it is vice versa if it is negative. The spreading coefficient of surfactant molecules on the surface of the sandstone was determined using Eq. (13).

13 $${{\rm{S}}_{{\rm{coff}}}}\,{\rm{ =\,}}{{\rm{\gamma }}_{\rm{L}}}\left( {{\rm{Cos}}\theta{\rm{ }} - 1} \right)$$

The spreading coefficient (Scoff) is equal to the difference between the work adhesion of liquid and its work of cohesion for the sandstone model.

14 $${{\rm{W}}_{\rm{a}}}{\rm{-}}{{\rm{W}}_{\rm{c}}}{\rm{\,=\,}}{{\rm{S}}_{{\rm{coff}}}}$$

Where Scoff is the spreading coefficient, γ_L_ is the IFT and θ is the contact angle (finite), (Cos θ ^−1^) is always negative and resulting S_coff_ negative value as shown in Fig. 5. If the contact angle is 0, the spreading coefficient may be zero or positive. In both cases, total spreading wetness, take place. It can be used spreading coefficient as an index to measure wettability because the spreading coefficient is positive and rises with increasing wettability^51,61^.

From the data in Table 4, it was found that, the spreading coefficient of the (PMRH 136 + RHATAS ) equal − 0.87 × 10 ^− 3^. Meanwhile, it was − 15 × 10^− 4^ for the individual surfactant PMRH136 .

### Surface charge energy

The surface-free energy was calculated using Eq. (15).

15 $${{\rm{\gamma }}_{\rm{L}}}\,{\rm{cos \,\theta = \theta \,}}{{\rm{\gamma }}_{\rm{S}}}\,{{\rm{\gamma }}_{{\rm{SL}}}}$$

Where γ_SL_ the interfacial tension of the system, γ_L_ the surface tension of the liquid (m N/m), and γ_S_ the surface free energy of the rock, by rearranging Eq. (16)^51^.

16 $${{\rm{\gamma }}_{\rm{S}}}{\rm{\,=\,}}{{\rm{\gamma }}_{\rm{L}}}\,{\rm{cos\, \theta /\;\theta\,}}{{\rm{\gamma }}_{{\rm{SL\;\;\;\;\;\;\;\;\;\;\;\;}}}}{\rm{mJ/}}{{\rm{m}}^{\rm{2}}}$$

The maximum value of the surface charge energy was obtained with the PMRH136 (44.64mJ/m^2^). Meanwhile the minimum value was achieved by the PMRH9 (0.66064mJ/m^2^ ). However, the lowest interfacial tension (4 × 10^− 2^ mNm^− 1^) was exhibited against the blend ( PMRH136 + RHATAS). As a result of decreasing the tension, this blend achieved a surface charge energy equal (72.68 mJ/m^2^). It can be concluded that the increase of ethereal bond (-o- ), increases the surface charge energy. The increase of oxygen bonds along the ethylene oxide chain in the surfactant molecules plays an important role in increasing the surface charge energy as shown in Fig. 5. This means that the surface charge energy is directly affected by the surface of the liquid and the contact angle, which provides that, the surfactant chemical structure is very important in the EOR.

### Rheological properties

The rheological properties of the synthesized nonionic surfactant at a salinity of 50.000 ppm were determined at 25 and 50°C. The Herschel-Bulk ley equation was utilized to analyze the flow curve^62^.

**Fig. 6:**
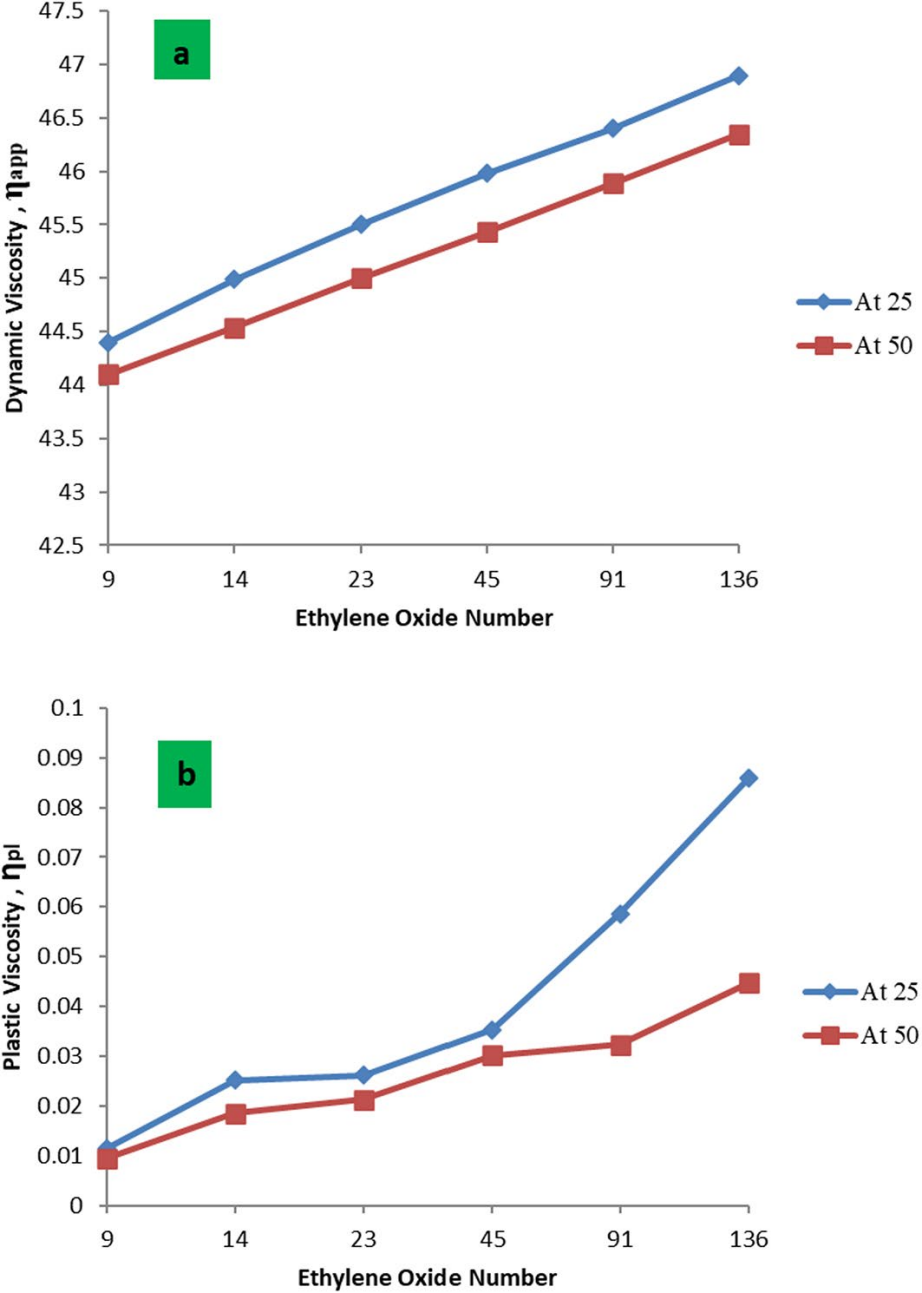
The relation between (**a**) η_app_ and (**b**) η_pla_Vs EO number at different temps.

**Fig. 7:**
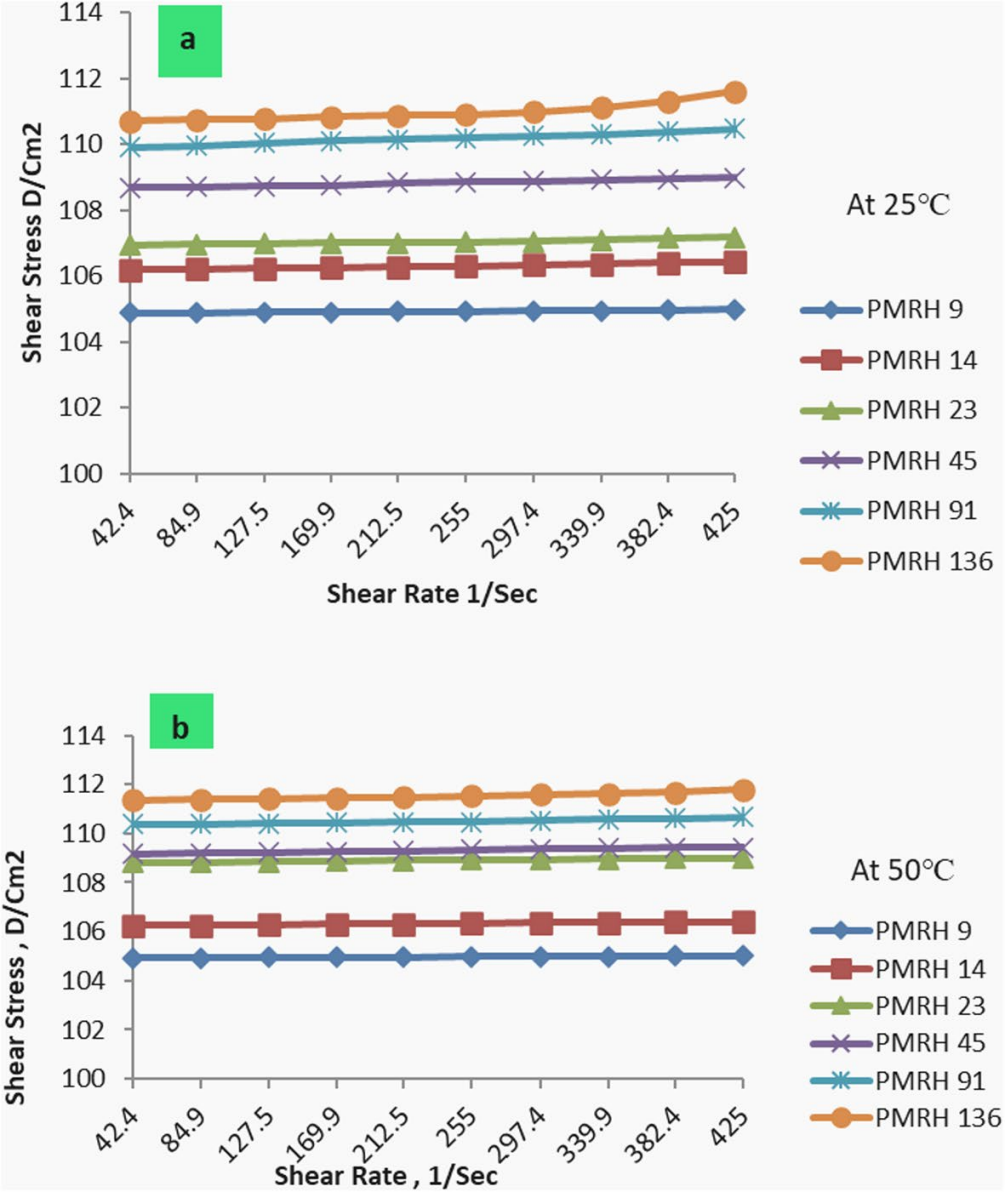
The relation between shear stress and shear rate at different temp.

The effect of the used nonionic surfactant on the rheological behavior of formation water at different temperatures was listed in Tables 5 and 6. It was found that the values of apparent viscosity ( η_app_ ) and plastic viscosity ( η_pl_ ) of the PMRH 9, PMRH 14, PMRH 23, PMRH 45, PMRH 91, PMRH 136 increase with increasing of ethylene oxide number and decrease with increasing temperature as shown in Fig. 6. The yield value (ƮB) increases with increased ethylene oxide number and temperature as shown in Table 5; Fig. 7.

One can conclude that these surfactants not only reduce the interfacial tension but also increase the viscosity of their solutions. This finding gives these surfactants the privilege to play two roles in the EOR process. The first role is to minimize the IFT, which serve to repel the emulsion-forming oil droplet. The second role is to increase the viscosity of the chemical flooding solution which may act as sweeping of oil and emulsion from the pores, consequently enhancing the recovery factor. This observation may be pronounced as the result of an increase of ethylene oxide chains that are coiling in the solution, consequently, the viscosity increases. Enhanced Oil Recovery Factor of the Surfactant Flooding.

The nonionic and cationic surfactants were used in chemical flooding formulations to assess the effectiveness of oil recovery. To change the wettability of the rock surface, the flooding approach depends on choosing the ideal surfactant concentration while accounting for variables including critical micelle concentration (CMC), interfacial tension (IFT), and contact angle (CA). These elements have a great impact on how the flooding process turns out.

The injected pore volume of a sand-packed model was used for various sets of flooding experiments for the nonionic and cationic surfactants at somewhat higher CMC concentrations, temperatures (50 °C), and salinities (50 × 10^3^ ppm). The trapped oil in the pores is released upon flooding with a surfactant solution due to a reduction in the IFT between the oil and the injecting surfactant solution. To interact with the trapped oil, it can lower the IFT, decrease the wettability of the rock, solubilize the oil by generating an oil-in-water emulsion, and raise the recovery factor (RF). By analysis of these factors, the (PMRH 9, PMRH 14, PMRH 23, PMRH 45, PMRH 91 PMRH 136, and RHATAS) exhibited high performance and favorable results. The process of flooding in a one-dimensional model with sandstone under reservoir conditions that were simulated was investigated through experiments. Two distinct methods—a secondary oil displacement (2ry recovery) and a tertiary oil displacement technique (3ry recovery), which involved two processes overall—were used to establish the oil recovery factor. After the sand pack was saturated with crude oil, brine was injected into it in the first stage, which is equivalent to the secondary recovery technique (2ry recovery). The remaining oil was left in the rock, but some of it was successfully extracted. High interfacial tensions alone—no chemical interaction—caused the oil to be displaced by the formation of water.

In order to recover the residual oil in the sand pack model, a tertiary recovery method (3ry recovery) has to be used in the second step. During the flooding process, this technique involves injecting solutions of surfactants (surfactants dissolved in formation water). These solutions reduced the interfacial tensions between the oil and formation water fluids and increased sweeping efficiency. The amount of recoverable oil rose as a result of increased ethylene oxide content. The oil recovery volume recovered in each step (secondary and tertiary oil displacement procedures) was added up to determine the total oil recovery factor (RF), which can be reported as a percentage in milliliters (ml) or as a percentage of the original oil in place (OOIP).

**Fig. 8:**
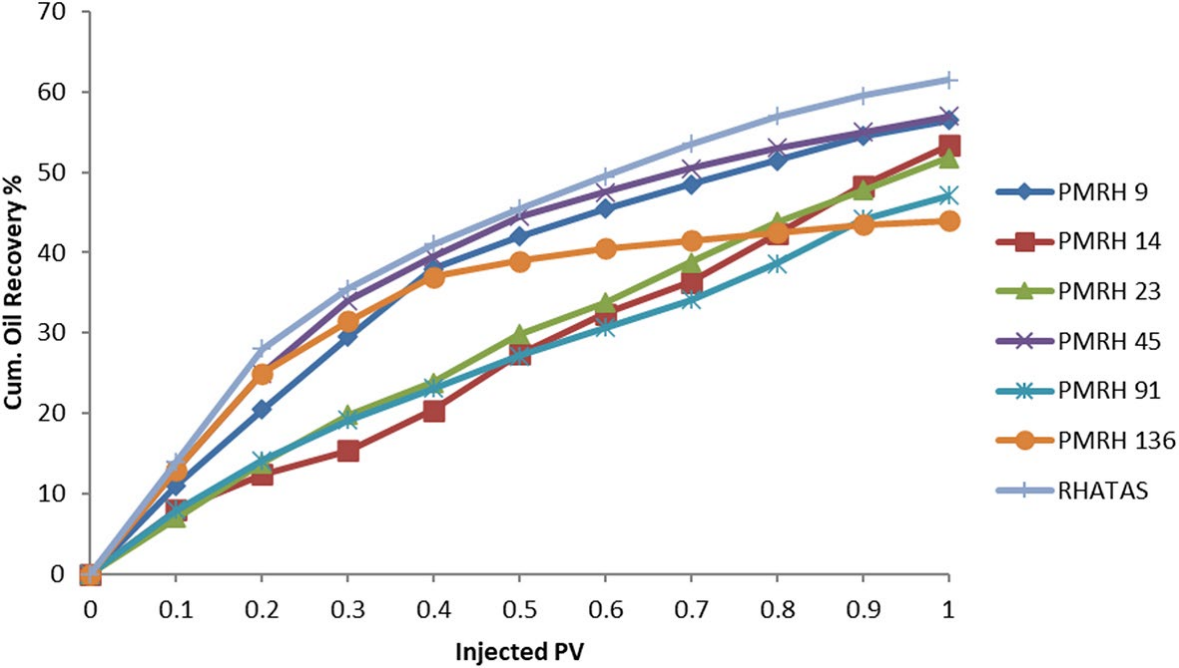
The cumulative oil at secondary recovery vs the injected pore volume.

**Fig. 9:**
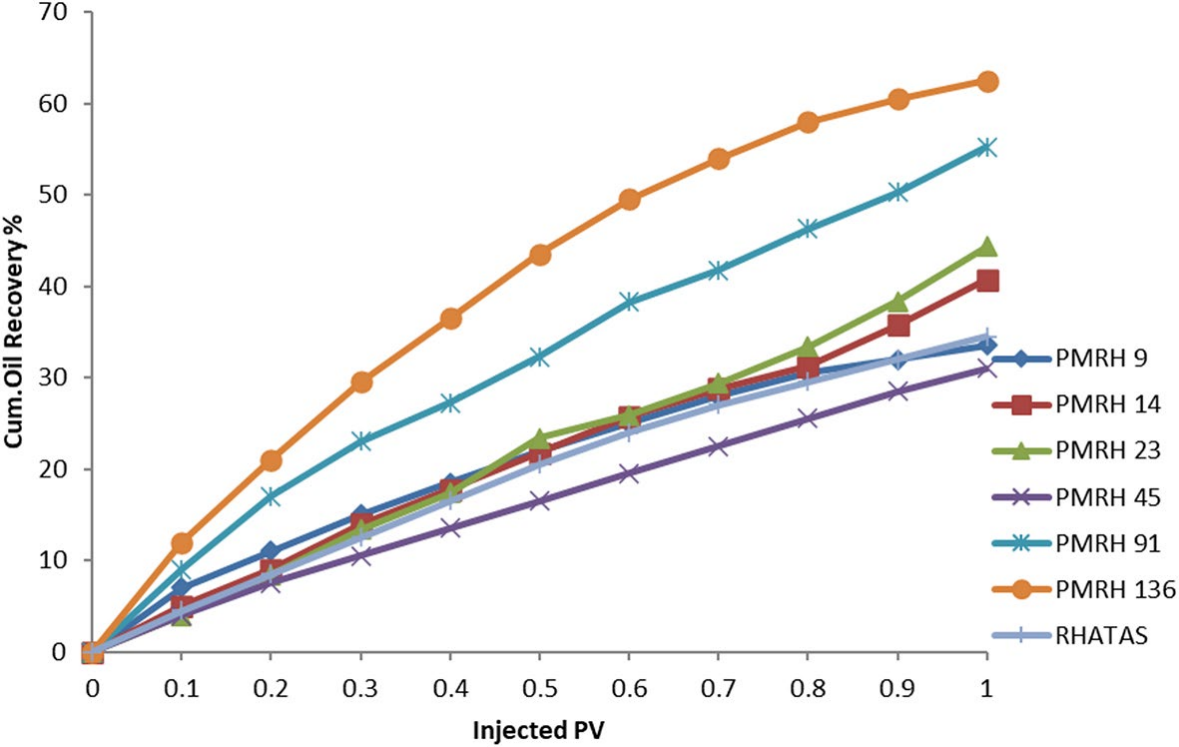
The cumulative oil at tertiary recovery vs the injected pore volume.

**Fig. 10:**
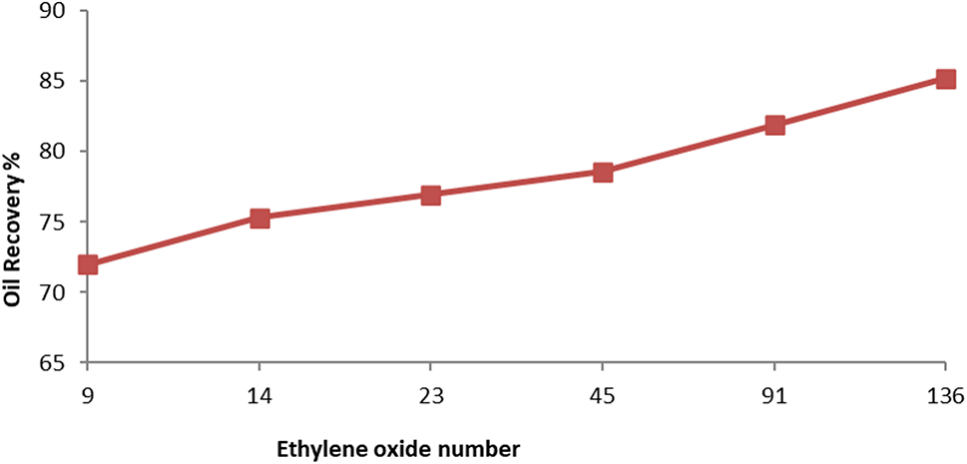
The relation between ethylene oxide number and recovery %.

**Fig. 11:**
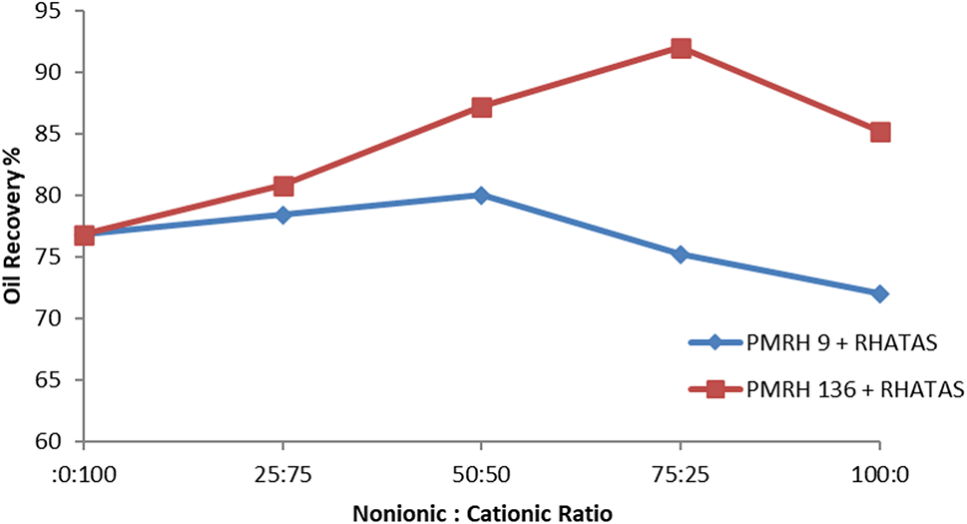
Relation between oil recovery and blend ratio.

The total recovery factor determined by secondary and tertiary recoveries is denoted by RF_Total_. According to results, the overall recovery factor for PMRH 9, PMRH 14, PMRH 23, PMRH 45, PMRH 91, PMRH 136, and RHATAS was 72%, 75.3%, and 76.94% OOIP, 78.6%, 81.9%, and 85.2% OOIP, respectively, and 76.8% OOIP for RHATAS as shown in Table 7.And Figs. 8 and 9. According to these results, the recovery factor increases as the ethylene oxide number increases as shown in (Fig. 10). The maximum recovery for individual used surfactant obtained PMRH 136 (85.20%), and the lowest recovery achieved by PMRH 9 (72%). However with blends between the RHATAS and PMRH 9 and with PMRH 136 at various ratios from (nonionic: cationic) surfactants, it was found that the blend (PMRH 136: RHATAS) pronounced the highest oil recovery factor at a ratio (75:25). As shown in Table 8; Fig. 11.The total recovery factor for this blend arrived 92%.

This result was obtained as the result of mixing surfactant at a certain ratio, a good micelle should be formed, and maximum reduction of IFT was achieved lowest contact angle. These factors make the maximum solubilization of the oil throughout the flooding procedure, furthermore, the RF increases.

**Table 5:** Dynamic viscosity parameters for the used surfactant at 25 and 50°C.

Temperature	25°C	50°C	25°C			50°C		
Sample	Viscosity CP		Shear Stress, D/Cm^2^	Shear Rate, 1/sec	Yeild Value ,ГB	Shear Stress, D/Cm^2^	Shear Rate, 1/sec	Yeild Value ,ГB
PMRH 9	246.90	246.90	104.88	42.40	0.02	104.92	42.40	0.01
	123.40	123.40	104.89	84.90		104.93	84.90	
	82.500	82.300	104.9	127.5		104.94	127.5	
	61.900	61.700	104.91	169.9		104.95	169.9	
	49.800	49.300	104.92	212.5		104.96	212.5	
	41.800	41.100	104.93	255.0		104.97	255.0	
	35.800	35.200	104.94	297.4		104.98	297.4	
	30.800	30.800	104.95	339.9		104.99	339.9	
	27.800	27.600	104.97	382.4		105.00	382.4	
	24.800	24.800	104.99	425.0		105.00	425.0	
PMRH 14	248.90	248.90	106.2	42.40	0.03	106.24	42.40	0.02
	125.90	125.90	106.22	84.90		106.26	84.90	
	82.900	82.500	106.24	127.5		106.28	127.5	
	62.890	62.230	106.26	169.9		106.30	169.9	
	50.880	50.400	106.28	212.5		106.32	212.5	
	42.600	41.900	106.31	255.0		106.34	255.0	
	36.300	35.900	106.34	297.4		106.36	297.4	
	31.600	30.800	106.37	339.9		106.38	339.9	
	27.900	27.800	106.40	382.4		106.39	382.4	
	24.900	24.800	106.42	425.0		106.39	425.0	
PMRH 23	250.99	250.99	106.94	42.40	0.04	108.82	42.40	0.03
	127.00	127.00	106.96	84.90		108.83	84.90	
	83.400	82.900	106.98	127.5		108.85	127.5	
	63.500	62.990	107.00	169.9		108.87	169.9	
	51.500	50.880	107.02	212.5		108.91	212.5	
	42.900	42.600	107.04	255.0		108.94	255.0	
	36.900	36.300	107.06	297.4		108.95	297.4	
	32.800	31.600	107.09	339.9		108.97	339.9	
	28.100	27.900	107.15	382.4		108.99	382.4	
	24.900	24.900	107.19	425.0		108.99	425.0	
PMRH 45	254.20	254.20	108.69	42.40	0.05	109.18	42.40	0.04
	131.00	131.00	108.71	84.90		109.21	84.90	
	83.900	83.200	108.73	127.5		109.23	127.5	
	63.800	63.200	108.75	169.9		109.25	169.9	
	51.900	51.400	108.84	212.5		109.28	212.5	
	42.900	42.800	108.86	255.0		109.33	255.0	
	37.600	36.900	108.89	297.4		109.36	297.4	
	33.400	32.900	108.93	339.9		109.39	339.9	
	28.800	28.200	108.95	382.4		109.42	382.4	
	25.600	24.900	108.99	425.0		109.44	425.0	
PMRH 91	256.80	256.80	109.91	42.40	0.06	110.37	42.40	0.05
	135.00	135.00	109.96	84.90		110.39	84.90	
	84.200	83.800	110.05	127.5		110.42	127.5	
	63.800	63.600	110.10	169.9		110.43	169.9	
	51.800	51.430	110.15	212.5		110.46	212.5	
	43.500	42.900	110.19	255.0		110.48	255.0	
	38.500	37.600	110.25	297.4		110.52	297.4	
	33.900	33.400	110.29	339.9		110.58	339.9	
	29.600	28.800	110.38	382.4		110.61	382.4	
	25.990	25.600	110.47	425.0		110.67	425.0	
PMRH 136	258.90	258.90	110.71	42.40	0.20	111.35	42.4	0,06
	136.50	136.50	110.74	84.90		111.39	84.9	
	84.900	84.200	110.78	127.5		111.43	127.5	
	63.890	63.500	110.83	169.9		111.45	169.9	
	52.500	51.600	110.87	212.5		111.49	212.5	
	43.990	43.550	110.91	255.0		111.54	255.0	
	38.900	38.500	110.99	297.4		111.58	297.4	
	34.600	33.900	111.12	339.9		111.62	339.9	
	29.900	29.600	111.30	382.4		111.68	382.4	
	26.600	25.990	111.60	425.0		111.79	425.0	

**Table 6:** Apparent and plastic viscosity at 25 and 50°C.

Surfactant	Apparent Viscosity, η_app_		Plastic Viscosity, η_pl_	
	25°C	50°C	25°C	50°C
PMRH 9	44.400	44.100	0.0115	0.0095
PMRH 14	44.990	44.540	0.0252	0.0185
PMRH 23	45.500	45.000	0.0262	0.0213
PMRH 45	45.980	45.430	0.0353	0.0302
PMRH 91	46.400	45.890	0.0586	0.0323
PMRH 136	46.900	46.350	0.0858	0.0447

**Table 7:** Cumulative oil recovery for PMRH 9, PMRH 14, PMRH 23, PMRH 45, PMRH 91, PMRH 136 and RHATAS.

Sample	Salinity (ppm)	Temperature (°C )	Sec. Recovery		Ter. Recovery		Residual oil	Total Recovery	
			CC	%	CC	%	CC	CC	%
PMRH 9	50.000	50	56.50	45.20	33.50	26.80	35.00	90.00	72.00
PMRH 14	50.000	50	53.38	40.75	40.75	35.50	30.88	94.13	75.30
PMRH 23	50.000	50	51.81	45.60	44.38	36.95	28.81	96.19	76.94
PMRH 45	50.000	50	50.25	44.38	48.00	38.40	26.75	98.25	78.60
PMRH 91	50.000	50	47.13	40.40	55.25	44.20	22.63	102.38	81.90
PMRH 136	50.000	50	44.00	35.20	62.50	50.00	18.50	106.50	85.20
RHATAS	50.000	50	61.50	49.20	34.50	27.60	29.00	96.00	76.80

**Table 8:** Effect of blends (Nonionic: Cationic) surfactants on enhanced oil recovery.

Blends	Ratio, %	Sec. Recovery		Ter. Recovery		Residual oil	Total Recovery	
		CC	%	CC	%	CC	CC	%
PMRH 9 + RHATAS ( Nonionic : Cationic)	0.0 : 100	61.5	49.2	34.5	27.6	29.0	96.0	76.8
	25 : 75	63.0	50.4	35.0	28.0	27.0	98.0	78.4
	50 : 50	64.6	51.7	35.4	28.3	25.0	100.0	80.0
	75 : 25	58.0	46.4	36.0	28.8	31.0	94.0	75.2
	100 : 0.0	56.5	45.2	33.5	26.8	35.0	90.0	72.0
PMRH 136 + RHATAS ( Nonionic : Cationic)	0.0 : 100	61.5	49.2	34.5	27.6	29.0	96.0	76.8
	25 : 75	47.0	37.6	54.0	43.2	24.0	101.0	80.8
	50 : 50	49.0	39.2	60.0	48.0	16.0	109.0	87.2
	75 : 25	39.0	31.2	76.0	60.8	10.0	115.0	92.0
	100 : 0.0	44.0	35.2	62.5	50.0	18.5	106.5	85.2

### Mechanism of enhanced oil recovery method^42^

**Fig. 12:**
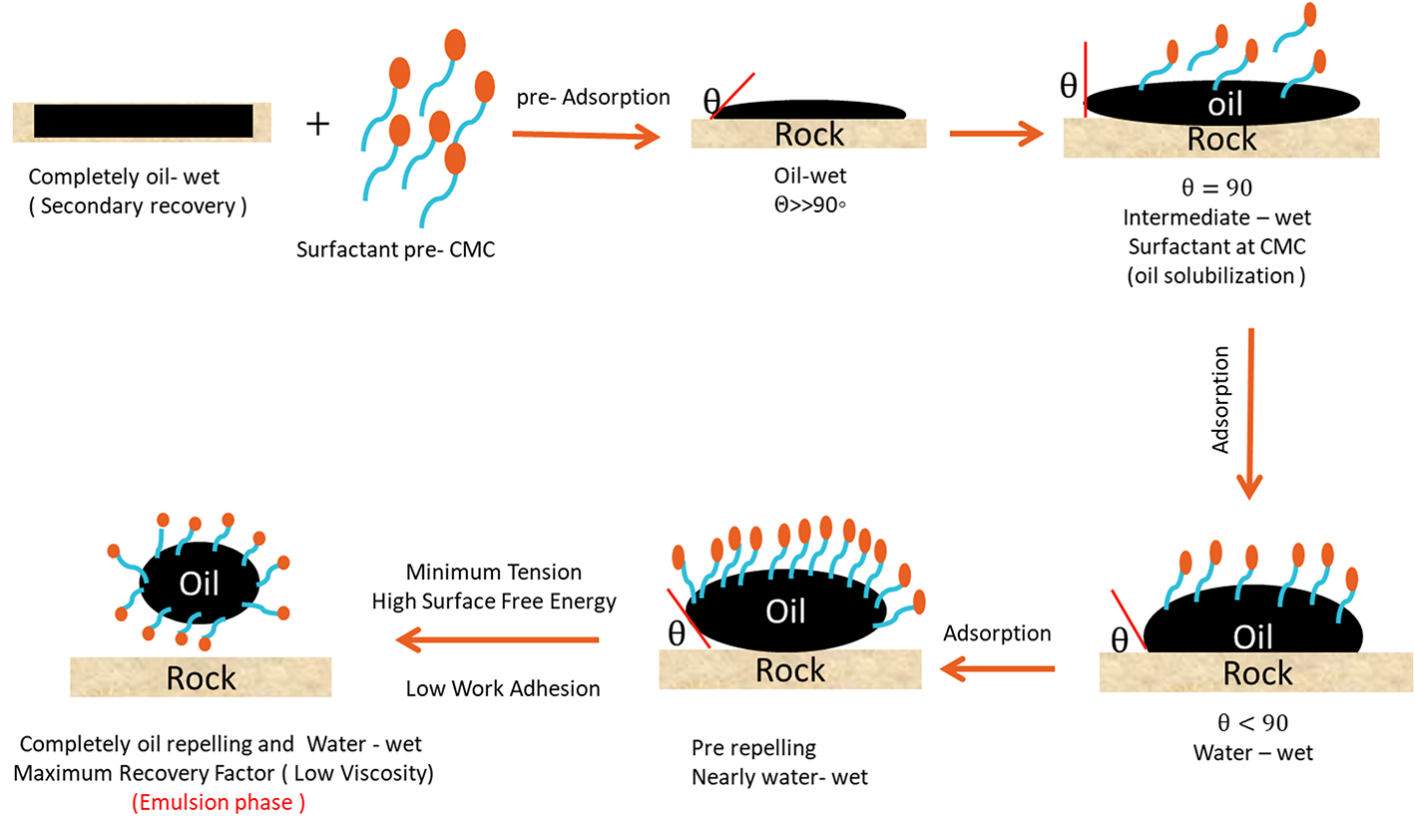
The mechanism of EOR.

In this work the mechanism of enhancing the recovery factor on the light of changing wettability, contact angle, IFT, work adhesion, spreading coefficient, and surface charge energy is described in different phases as shown in Fig. 12. At the surfactant phase, the rock (sand backed model) is completely oil-wet in the presence of surfactant, the molecules are adsorbed continuously up to make completely adsorption which have been achieved at the CMC. The adsorption process passes via the adsorption of monomeric molecules, then at the pre-micelle phase up to the complete micelle form. The oil droplet is in complete contact with the oil-wet rock surface, by introducing the surfactant pre-CMC will affect the contact angle, where, the rock surface that is moist with oil has the oil droplet adhered to it (θ>>90˚). At the solubilization phase, the trapped oil surface is surrounded by a monomeric adsorbed layer created by the surfactant (on the surface of oil, surfactant molecules begin to adsorb ) at this point the surface is still oil wet but the contact angle somewhat reduces ( θ = 90˚), the complete monolayer of the adsorbed surfactant is formed at the CMC of the surfactant. The surfactant molecules reduce the IFT by penetrating the rock-oil contact. Therefore, by forming a persistent layer between the rock surface and the oil droplet, the penetrating surfactant molecules reduce the contact angle further (θ ≤ 90˚), which is called intermediate wettability. At pre- repelling phase ( nearly water-wet ), The IFT and work adhesion will reach to minimum value and high surface free energy at the final stage, and the contact angle is going very low (θ << 90˚), The oil droplets are going to complete repelling and the sand backed model ( rock ) is nearly water - wet. After that, the O/W emulsion is formed, which is swept easily through the pore during the adsorption of surfactant molecules to achieve maximum recovery factor.

## Conclusion

The conclusion of this work can be addressed in the following points.

- The γ_min_ and (n _min_ ) (alkane carbon number, ACN) at 50°C and CMC concentration were determined by the n-hydrocarbon scans (n-c6 to n-c18) against the interfacial tension. From the obtained result, it was found that the γ_min_ recorded in order of 10^− 2^ mNm ^− 1^ at ( n _min_ ) n-c14.
- The work adhesion (W_a_), surface charge energy, and spreading coefficient were calculated for the used surfactants on the basis of surface interfacial tension and contact angle.
- The rheological properties of the solution of these surfactants at CMC and 50 °C were measured to determine the dynamic (η_app_) Bingham yield value and plastic viscosity (η_pl_). These surfactants exhibited a reduction in IFT and they increased the η_app_ and η_pl_ viscosity of their solution, which gives the privilege to apply in the EOR.
- The W_a_, γ_s_, and W_s_ were very sensitive to the increase of ethylene oxide number of surfactant. The W_a_ decreased, the γ_s_ increased and the W_s_ also increased by the increase of EO units in the surfactant molecules. The recovery factor was directly affected by these factors.
- The recovery factor of the crude oil was investigated by using a sand back model at 50 °C on crude oil (API (35), Pour Point (9), and Wax Content (15.5%).
- From the obtained data it was found that PMRH 136 exhibited the maximum oil recovery (85.20%), meanwhile, its blend with RHATAS exhibited a recovery factor (92%).

## Electronic supplementary material

Below is the link to the electronic supplementary material.

Supplementary Material 1

## Data Availability

The data used and analyzed during the current study are available from the corresponding author upon reasonable request as long as the request does not compromise intellectual property interests.
