# Supplementary material for: Crude oil recovery by new nonionic surfactants in relation to equivalent alkane carbon number (EACN) with work adhesion and alteration wettability
Source: Sci Rep. 2025 May 17;15:17166. doi: 10.1038/s41598-025-99993-8 (PMC12085648; doi:10.1038/s41598-025-99993-8)
Supplement: Supplementary file 1 — Supplementary Material 1 [file 41598_2025_99993_MOESM1_ESM.doc]

**Fig.1: Chromatogram of Crude Oil Sample**

Table1: Gas chromatogram of Oil Sample

| **Crude Oil Sample** | | | | | |
| --- | --- | --- | --- | --- | --- |
| **Gas chromatographic analysis of Oil Sample** | | | | | |
| **G-2022-347** | | | | | |
| **31/08/2022** | | | | | |
| **Components** | **Molecular Structure** | **Carbon No.** | **Wt. %** | **Mol. Fraction%** | **No. of mole =**  **Mol fraction / M.Wt** |
| **Ethane** | **C_2_H_6_** | **C_2_** | **0.000** | **0.000** | **0.000** |
| **Propane** | **C_3_H_8_** | **C_3_** | **0.000** | **0.000** | **0.000** |
| **i-Butane** | **HC(CH_3_)_3_** | **i-C_4_** | **0.093** | **0.312** | **0.537** |
| **n-Butane** | **C_4_H_10_** | **n-C_4_** | **0.252** | **0.843** | **1.453** |
| **i-pentane** | **CH_3_CH_2_CH(CH_3_)_2_** | **i-C_5_** | **1.173** | **3.156** | **4.383** |
| **n-Pentane** | **C_5_H_12_** | **n-C_5_** | **1.023** | **2.754** | **3.825** |
| **Hexanes** | **C_6_H_14_** | **C_6_** | **9.225** | **24.78** | **28.81** |
| **Heptanes** | **C_7_H_16_** | **C_7_** | **12.72** | **25,76** | **25.76** |
| **Octane** | **C_8_H_18_** | **C_8_** | **19.49** | **34.67** | **30.41** |
| **Nonanes** | **C_9_H_20_** | **C_9_** | **17.35** | **26.28** | **20.53** |
| **Decanes** | **C_10_H_22_** | **C_10_** | **15.41** | **21.04** | **14.81** |
| **Undecanes** | **C_11_H_24_** | **C_11_** | **14.24** | **17.69** | **11.34** |
| **Dodecanes** | **C_12_H_26_** | **C_12_** | **13.15** | **14.99** | **8.821** |
| **Tridecanes** | **C_13_H_28_** | **C_13_** | **12.39** | **13.05** | **7.094** |
| **Tetradecanes** | **C_14_H_30_** | **C_14_** | **11.86** | **11.61** | **5.863** |
| **Pentadecanes** | **C_15_H_32_** | **C_15_** | **11.04** | **10.10** | **4.764** |
| **Hexadecanes** | **C_16_H_34_** | **C_16_** | **10.70** | **9.183** | **4.063** |
| **Heptadecanes** | **C_17_H_36_** | **C_17_** | **12.82** | **10.35** | **4.316** |
| **Octadecanes** | **C_18_H_38_** | **C_18_** | **11.61** | **10.20** | **4.015** |
| **Nonadecanes** | **C_19_H_40_** | **C_19_** | **11.17** | **8.397** | **3.133** |
| **Icosanes** | **C_20_H_42_** | **C_20_** | **10.56** | **7.680** | **2.723** |
| **Eneicosanes** | **C_21_H_44_** | **C_21_** | **10.23** | **6.921** | **2.338** |
| **Dodeicosanes** | **C_22_H_46_** | **C_22_** | **9.945** | **6.402** | **2.065** |
| **Tricosanes** | **C_23_H_48_** | **C_23_** | **9.666** | **5.949** | **1.836** |
| **Tetraicosanes** | **C_24_H_50_** | **C_24_** | **8.805** | **5.544** | **1.640** |
| **Petaicosanes** | **C_25_H_52_** | **C_25_** | **8.208** | **4.848** | **1.377** |
| **Hexaicosanes** | **C_26_H_54_** | **C_26_** | **7.269** | **4.347** | **1.187** |
| **Heptaicosanes** | **C_27_H_56_** | **C_27_** | **6.678** | **3.708** | **0.975** |
| **Octaicosanes** | **C_28_H_58_** | **C_28_** | **5.721** | **3.285** | **0.833** |
| **Nonaicosanes** | **C_29_H_60_** | **C_29_** | **5.049** | **2.718** | **0.666** |
| **Tricontanes** | **C_30_H_62_** | **C_30_** | **4.188** | **2.319** | **0.549** |
| **Entricontanes** | **C_31_H_64_** | **C_31_** | **3.759** | **1.857** | **0.425** |
| **Dodetricontanes** | **C_32_H_66_** | **C_32_** | **2.958** | **1.620** | **0.360** |
| **Tritricontanes** | **C_33_H_68_** | **C_33_** | **2.334** | **1.236** | **0.266** |
| **Tetratricontanes** | **C_34_H_70_** | **C_34_** | **1.632** | **0.945** | **0.197** |
| **Pentatricontanes** | **C_35_H_72_** | **C_35_** | **1.203** | **0.642** | **0.130** |
| **Hexatricontanes** | **C_36_H_74_** | **C_36_** | **0.957** | **0.462** | **0.091** |
| **Hepatricontanes** | **C_37_H_76_** | **C_37_** | **0.636** | **0.357** | **0.068** |
| **Octatricontanes** | **C_38_H_78_** | **C_38_** | **0.375** | **0.231** | **0.043** |
| **Nonatricontanes** | **C_39_H_80_** | **C_39_** | **0.278** | **0.132** | **0.024** |
| **Tetracontane plus** | **C_40_H_82_** | **C_40+_** | **0.639** | **0.213** | **0.037** |
| **Total** |  |  |  |  | **199.41** |
| **Total Mol.Wt.** | **199.41** | | | | |
| **HC %** | **33.27** | | | | |
| **UCM %** | **66.73** | | | | |

Mol.Wt. (Of C14 = H _n_ C _n +2_ = 12×14 + 30) = 198.

It is nearly equivalent of GC total molecular weight ( 199.41 ).

This mean that the EACN equivalent n-C_14_

Fig .2: Scheme of Synthesis of Non Ionic Surfactant^1^

Fig .3: Scheme of Synthesis of Gemini Cationic Surfactant^1^

Table2: Original data for IFT and ACN

| Surfactant | IFT | log IF | (n-alkane carbon number) |
| --- | --- | --- | --- |
|  | 1.0×10^-1^ | -1.000 | 6 |
|  | 2.5×10^-1^ | -0.602 | 8 |
|  | 5.0×10^-1^ | -0.301 | 10 |
| PMRH 9 | 7.0×10^-1^ | -0.154 | 12 |
|  | 9.0×10^-1^ | -0.045 | 14 |
|  | 5.5×10^-1^ | -0.259 | 16 |
|  | 3.0×10^-1^ | -0.522 | 18 |
|  | 0.7×10^-1^ | -1.154 | 6 |
|  | 2.0×10^-1^ | -0.698 | 8 |
|  | 3.2×10^-1^ | -0.494 | 10 |
| PMRH 14 | 5.0×10^-1^ | -0.301 | 12 |
|  | 7.0×10^-1^ | -0.154 | 14 |
|  | 3.5×10^-1^ | -0.455 | 16 |
|  | 0.9×10^-1^ | -1.045 | 18 |
|  | 5.5×10^-2^ | -1.254 | 6 |
|  | 1.0×10^-1^ | -1.000 | 8 |
|  | 2.0×10^-1^ | -0.698 | 10 |
| PMRH 23 | 3.5×10^-1^ | -0.455 | 12 |
|  | 5.0×10^-1^ | -0.301 | 14 |
|  | 2.5×10^-1^ | -0.702 | 16 |
|  | 5.5×10^-2^ | -1.258 | 18 |
|  | 2.5×10^-2^ | -1.602 | 6 |
|  | 6.0×10^-2^ | -1.221 | 8 |
|  | 9.9×10^-2^ | -1.004 | 10 |
| PMRH 45 | 1.9×10^-1^ | -0.721 | 12 |
|  | 3.0×10^-1^ | -0.522 | 14 |
|  | 9.7×10^-2^ | -1.009 | 16 |
|  | 3.7×10^-2^ | -1.423 | 18 |
|  | 1.5×10^-2^ | -1.812 | 6 |
|  | 2.5×10^-2^ | -1.602 | 8 |
|  | 4.5×10^-2^ | -1.346 | 10 |
| PMRH 91 | 1.1×10^-1^ | -0.954 | 12 |
|  | 1.9×10^-1^ | -0.700 | 14 |
|  | 7.5×10^-2^ | -1.122 | 16 |
|  | 2.3×10^-2^ | -1.620 | 18 |
|  | 1.0×10^-2^ | -2.000 | 6 |
|  | 1.8×10^-2^ | -1.700 | 8 |
|  | 3.0×10^-2^ | -1.500 | 10 |
| PMRH 136 | 5.2×10^-2^ | -1.280 | 12 |
|  | 1.2×10^-1^ | -0.915 | 14 |
|  | 3.1×10^-2^ | -1.497 | 16 |
|  | 3.5×10^-2^ | -1.950 | 18 |
|  | 0.7×10^-2^ | -2.154 | 6 |
|  | 1.1×10^-2^ | -1.958 | 8 |
|  | 2.0×10^-2^ | -1.698 | 10 |
| RHATAS | 3.9.0×10^-2^ | -1.400 | 12 |
|  | 9.0×10^-2^ | -1.045 | 14 |
|  | 2.5×10^-2^ | -1.590 | 16 |
|  | 1.0×10^-2^ | -2.000 | 18 |
|  | 0.2×10^-2^ | -2.698 | 6 |
|  | 0.7×10^-2^ | -2.154 | 8 |
| PMRH 9  + | 1.1×10^-2^ | -1.958 | 10 |
| RHATAS | 2.5×10^-2^ | -1.602 | 12 |
|  | 5.0×10^-2^ | -1.300 | 14 |
|  | 1.0×10^-2^ | -2.000 | 16 |
|  | 0.3×10^-2^ | -2.522 | 18 |
|  | 0.03×10^-2^ | -3.522 | 6 |
|  | 0.1×10^-2^ | -3.000 | 8 |
| PMRH 136  + | 0.2×10^-2^ | -2.698 | 10 |
| RHATAS | 0.5×10^-2^ | -2.301 | 12 |
|  | 1.0×10^-2^ | -2.000 | 14 |
|  | 0.3×10^-2^ | -2.522 | 16 |
|  | 0.12×10^-2^ | -2.920 | 18 |

**
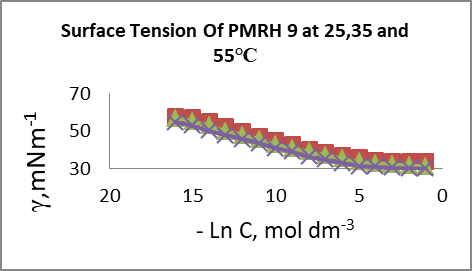

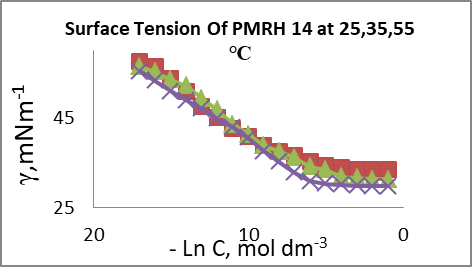
**

**
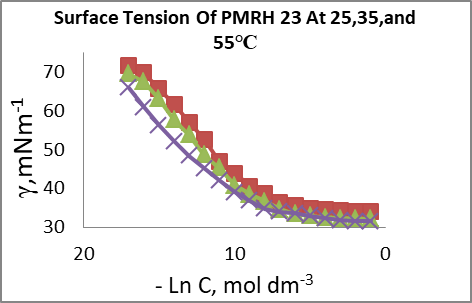

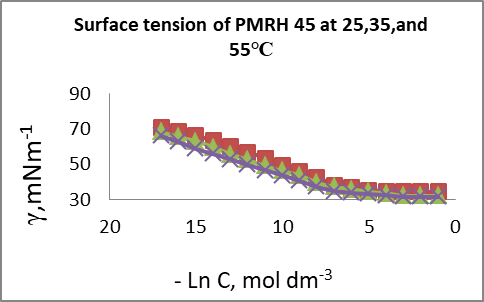
**

**
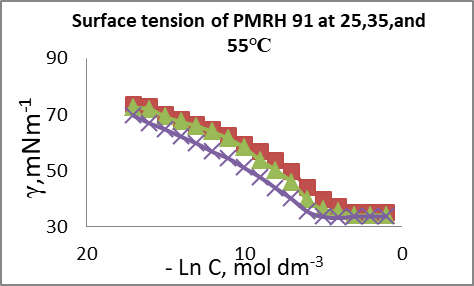

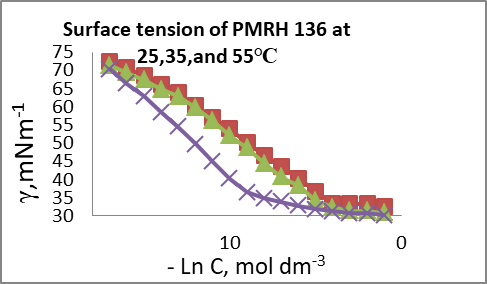
**

**
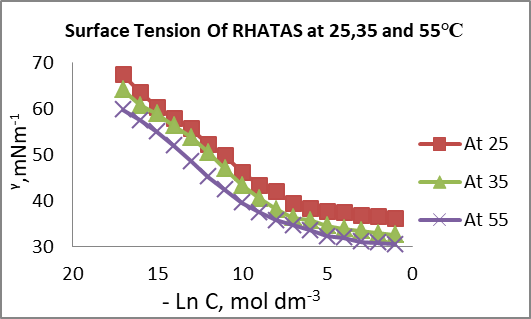
**

**Fig.4 : γ –lnC for PMRH and RHATAS at different temp.( ■ At 25℃ , ▲ At 35 , × At 55℃ )**^1^

**Table 3: Surface active properties of poly alkylene oxide maleate ester amide of ricinoleic hydrazide (PMRH x) and bis, ricinoleic hydrazide amide of N, N, N`, N` tetra methyl ethylene di amine ammonium salt Surfactant (RHATAS)**^1^

| **sample** | **Temp. °C** | **CMC,mol. dm^-3^×10^-3^** | **Υ_CMC_ , mN m^-1^** | ***P*C_20_ , mol.dm^-3^** | **π_CMC_ , mNm^-1^** | **Γ_max_  , mol/cm^2^**  **× 10^-10^** | **A_min_ , nm^2^  × 100** |
| --- | --- | --- | --- | --- | --- | --- | --- |
| **PMRH 9**  **Eo.no 9** | 25 | 6.56 | 34.25 | 1.05 | 23.42 | 2.40 | 0.692 |
|  | 35 | 3.15 | 33.66 | 1.08 | 23.12 | 2.90 | 0.573 |
|  | 55 | 1.58 | 32.00 | 1.10 | 22.89 | 3.40 | 0.489 |
| **PMRH 14**  **Eo.no 14** | 25 | 4.16 | 34.48 | 1.09 | 23.11 | 2.10 | 0.722 |
|  | 35 | 2.07 | 34.11 | 1.06 | 22.34 | 2.60 | 0.638 |
|  | 55 | 1.03 | 32.76 | 1.11 | 22.22 | 3.10 | 0.534 |
| **PMRH 23**  **Eo.no 23** | 25 | 2.22 | 36.26 | 1.068 | 35.44 | 2.13 | 0.791 |
|  | 35 | 1.43 | 36.60 | 1.093 | 32.95 | 2.30 | 0.722 |
|  | 55 | 0.66 | 36.80 | 1.11 | 29.10 | 2.50 | 0.664 |
| **PMRH 45**  **Eo.no 45** | 25 | 1.56 | 36.82 | 1.04 | 34.16 | 1.91 | 0.874 |
|  | 35 | 1.09 | 37.21 | 1.06 | 31.22 | 2.10 | 0.791 |
|  | 55 | 0.37 | 37.14 | 1.09 | 29.19 | 2.40 | 0.692 |
| **PMRH 91**  **Eo.no 91** | 25 | 1.14 | 37.18 | 0.98 | 36.37 | 1.77 | 0.938 |
|  | 35 | 0.74 | 36.00 | 0.99 | 36.47 | 1.91 | 0.870 |
|  | 55 | 0.23 | 35.36 | 1.06 | 34.08 | 2.20 | 0.755 |
| **PMRH 136**  **Eo.no 136** | 25 | 0.78 | 33.50 | 1.04 | 38.83 | 1.65 | 1.006 |
|  | 35 | 0.55 | 34.19 | 1.05 | 37.16 | 1.91 | 0.874 |
|  | 55 | 0.15 | 34.80 | 1.10 | 35.50 | 2.20 | 0.755 |
| **RHATAS** | 25 | 0.05 | 38.30 | 1.02 | 29.11 | 1.73 | 0.810 |
|  | 35 | 0.24 | 36.33 | 1.03 | 27.67 | 1.89 | 0.870 |
|  | 55 | 0.45 | 35.78 | 1.07 | 25.22 | 2.05 | 0.960 |
